# Serosurvey of Tick-Borne Encephalitis Virus Infection in Romania

**DOI:** 10.3390/pathogens13030231

**Published:** 2024-03-06

**Authors:** Andreea Mădălina Panciu, Cristina Alexandra Cheran, Eliza Daniela Militaru, Claudia Doina Rîciu, Adriana Hristea

**Affiliations:** 1 “Prof. Dr. Matei Bals” National Institute of Infectious Diseases, 021105 Bucharest, Romania; cristina-alexandra.constantin@drd.umfcd.ro (C.A.C.); eliza.manea@drd.umfcd.ro (E.D.M.); laboratorvirusologie@yahoo.com (C.D.R.); adriana.hristea@umfcd.ro (A.H.); 2 Faculty of Medicine, Carol Davila University of Medicine and Pharmacy, 020021 Bucharest, Romania

**Keywords:** TBEV, serosurvey, Romania

## Abstract

Background: Tick-borne encephalitis (TBE) is a disease with mandatory declaration in the EU since 2012. Information regarding the seroprevalence of the disease across Romania is limited, and only sporadic cases are rarely reported. We aimed to identify new areas of TBEV infection in different counties of Romania. Methods: We conducted a serosurvey assessing the immune response to TBEV infection in adult populations from rural areas in different counties of the country. Seropositivity was defined by a positive TBEV IgM/IgG ELISA test and confirmed by serum neutralization. Results: We collected 1116 samples from 15 different localities in 10 counties (divided into endemic/border/non-endemic counties) across Romania. Overall, 26 (2.3%) of the samples were tested positive using the TBEV ELISA assay in six counties. In those counties, 3.7% of sera were positive, varying from 1.4% to 6.9% per county. After performing the neutralization assay, seven (0.6%) samples were confirmed positive, interestingly all from one site in Sibiu County, where the seroprevalence was 9.7%. Conclusions: The identification of positive serum samples in serosurveys appears to rely on the discovery of TBEV microfoci. Further serological surveys should be conducted in Romania, especially after the identification of positive TBEV patients presenting for medical care.

## 1. Introduction

Tick-borne encephalitis (TBE) is an infectious disease caused by the tick-borne encephalitis virus (TBEV), a member of the family *Flaviviridae* [1]. 

There is currently no precise definition of the geographical distribution of TBEV in Europe. Nevertheless, the hard tick *Ixodes ricinus*, regarded as the main vector, is the most frequent species of tick in Romania. In a study where 13,771 ticks were collected from all 41 Romanian counties, *I. ricinus* was present in 97.7% of the 188 locations studied, and it was the dominant species of ticks [2].

In comparison with the widespread distribution of *I. ricinus*, TBEV circulation is limited to so-called “natural foci”, which are regions that can be as small as 50 × 50 m and are defined as areas of active virus transmission. Detecting such foci by screening ticks can be difficult due to the low TBEV prevalence in the tick population, even within natural foci [3]. In Romania, TBEV was identified in vector arthropods. Thus, in a study published in 2009, analyzing vector arthropods collected between 1985 and 1993, nine TBEV isolates were obtained from ticks collected from farm animals in five counties (Hunedoara, Tulcea, Mures, Alba, Caras-Severin) [4]. In another study, in three Romanian counties (Sibiu, Tulcea, Giurgiu) selected as tick sampling sites, specific RNAs from TBEV (3′ UTR- genomic region) were detected in <1% of *I. ricinus* in the ticks collected from vegetation, livestock and reptiles [5].

Assessing animal exposure to TBEV can be more informative than screening ticks regarding TBEV distribution and their associated public health risk. A significant correlation between TBE incidence in humans and seroprevalence in sentinel species has been shown in endemic areas [4]. In Romania, TBEV antibodies were detected by a serum neutralization assay in samples from 519 sheep from 50 localities in five counties in North-West Romania (Bihor, Bistrita-Nasaud, Cluj, Mures, Salaj). The total seroprevalence rate was 15.2%, with ranges from 2.0% to 27.7%, and our country has the third largest sheep flock in the European Union [6].

Although in Romania *I. ricinus* ticks have a large distribution, they have been found on a variety of domestic animals, and infected animals with TBEV have been found, the risk of TBE is reported mainly in Transylvania; however, data are scarce [7,8]. 

Between 2008 and 2014, a passive surveillance system based on reported cases, at a national level, of meningitis, encephalitis, meningoencephalitis, encephalomyelitis and encephalo-radiculitis tested for TBEV was implemented in Romania in the north-western counties around Cluj (Alba, Bihor, Bistrita-Nasaud, Cluj, Covasna, Harghita, Mures, Maramures, Satu Mare, Salaj, Sibiu, Arad, Hunedoara, Caras-Severin) [9]. Nevertheless, there is no regular screening, and the relative risk of contracting this disease is largely unknown. Most probably, the incidence of TBEV is underestimated [10].

We aimed to obtain information on the distribution and epidemiology of TBEV subclinical infection in counties not only where cases of TBEV have been reported but also in other areas known as non-endemic but where *I. ricinus* has been identified as the dominant species of ticks. Also, we chose rural areas since the population might be at a higher risk of the disease than those living in urban areas (a fact that may be caused by differences in tick habitat and exposure risk, not only due to outdoor activities but also by a lack of awareness and preventive measures in rural areas) [11].

## 2. Materials and Methods

We performed a cross-sectional serosurvey to test the immune response to TBEV infection in populations from different counties in Romania using a convenient sampling strategy. The sampling regions included endemic counties (with reported disease cases—Alba, Sibiu, Hunedoara), counties bordering endemic regions (Suceava, Neamt, Prahova, Brasov) and non-endemic counties (without any reported disease cases, not under passive surveillance, remote from endemic regions—Teleorman, Botosani, Constanta). 

### 2.1. Blood Sampling and Data Collection

**Figure 1:**
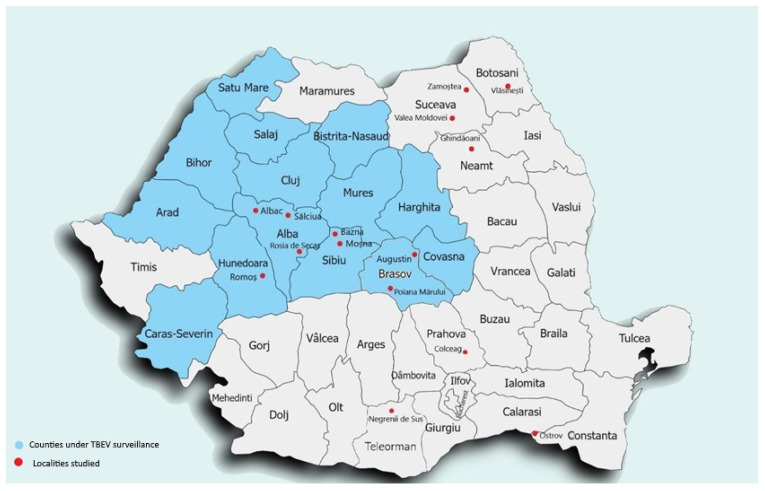
Serum sample localities where serum samples were collected (red dots) and counties under passive surveillance (blue).

The serum samples were collected during a two-year period (2021–2022). During this time, in 15 different localities in Romania., a total of 1116 samples from 10 counties in Romania were collected. Three of the counties were already under TBE surveillance, and cases of TBEV infection were reported between 2008 and 2013 (Alba, Sibiu, Hunedoara), and seven counties were not under epidemiological surveillance (Teleorman, Brasov, Suceava, Botosani, Neamt, Constanta, Prahova) (Figure 1).

To collect serum samples, we joined a medical mobile unit offering blood routine tests to people living in various remote rural sites. The different number of participants from each site reflects the number of volunteer participants in our study. After informed consent, the participants were interviewed using a paper-based questionnaire, which included demographic data such as profession, tick bite history, the presence of household animals and knowledge about diseases spread by tick bites; their blood was also drawn. 

A blood draw of 5 mL (in a whole blood tube—SST II Advance BD 5 mL) was performed by a certified nurse, following all aspects of patient safety. The blood sample necessary for this study was collected during the blood draw for routine tests, causing no additional physical or psychological harm to the subjects. There were no expenses for the patient for any assessment. The blood samples were centrifuged within the next 12 h after collection, and the sera were frozen at −20 °C within 2 h after centrifugation and stored at −80 °C.

### 2.2. Enzyme-Linked Immunosorbent Assay (ELISA) to Detect Anti-TBEV Antibodies

Serum samples were tested for TBEV-specific IgM and IgG antibodies using the commercial VIROTECH FSME/TBE IgG/IgM ELISA kit (VIROTECH Diagnostics GmbH, Russelsheim, Germany) according to the manufacturer’s instructions. Results were expressed as VIROTECH Units (VE), with VE < 9.0 considered negative, VE = 9.0–11.0 as borderline and VE > 11.0 as positive. The optical density of the ELISA plates was read using an automated analyzer, ELISA-EVOLIS (BIO-RAD, Hercules, CA, USA), at 450 and 620 nm. The specificity of the test is 95.6% for IgG and >99.8% for IgM for the TBE ELISA [12].

### 2.3. Neutralization Assay

Samples with an ELISA result of ≥9.0 VE were further tested by a serum neutralization test (SNT) to exclude cross-reactivity with other flavivirus infections. The SNT was performed as a micro-neutralization test. The test procedure is divided into three phases: the propagation of non-neutralized viruses in VERO cell culture, the detection of viral antigens by ELISA and the analysis of the results. The propagation of non-neutralized viruses in the VERO cell culture was performed by serial dilutions of test samples incubated with a constant amount of TBEV and subsequently inoculated on VERO cells grown in microtiter plates. A serum sample was considered positive for TBEV if the cells were protected at least at a serum dilution of >1:10. ELISA was performed using ELISA plates (NUNC Maxisorp, Thermo Fisher Scientific, Orth, Austria) coated with TBE-specific antibodies (guinea pig) for the capture of TBEV/antigen. The cell culture supernatant from the propagation step was transferred onto the ELISA plates. The bound antigen was detected by a second TBEV-specific antibody (rabbit) and an HRP-labeled anti-rabbit antibody (donkey). The data analysis was performed by determining the neutralizing titer of the sample according to the Kärber formula. Only samples from participants unvaccinated against yellow fever were analyzed. 

## 3. Results

### 3.1. Study Population

Overall, we collected a total of 1116 serum samples from 15 localities in 10 counties in Romania (Table 1). The median age of the participants was 58 (standard deviation: 14.4 years). The younger group, under <40 years, represented 13.7%; the group 40–55 years represented 29.9% of the study population; the older group >56 years, 56.3%. Most participants, 825 (74%), were female. 

Regarding known risk factors for TBEV infection, we found that a total of 231 (20.7%) participants reported having a profession at risk for tick bites (hunters, forest rangers, farmers, leather manufacturers). A popular activity in different regions of Romania is mushroom collecting, which gives great skin contact with green spaces, and people often mentioned in our conversations the frequent presence of “forest fleas”, as they named the ticks. From the study population, 266 (23.8%) reported mushroom collecting during the summer–spring season. As expected, as much as half of the participants reported consumption of unprocessed milk and milk products from their own production. 

Regarding the history of tick bites, there were 274 (24.5%) participants who reported having a tick bite history, 252 (22.5%) of them only once or a few times in their lives. Only 53 (4.7%) reported seeking medical attention for the tick bites. A total of 139 (12.4%) participants reported domestic animals having tick infestations (sheep, goats, horses). There were also 23 (2%) participants who reported having a history of CNS (central nervous system) symptoms. A total of 58 (5.2%) participants mentioned having leisure recreational activities in nature, like camping and trekking.

Knowledge regarding tick-borne diseases and precautions to be taken was reported by 834 (74.7%) participants. After completion of the questionnaires and blood sampling, all patients received flyers with information regarding tick-borne diseases and prevention methods, raising awareness regarding the topic. None of the participants were vaccinated for TBEV or yellow fever.

### 3.2. Anti-TBEV Antibody ELISA Results 

Overall, 26 (2.3%) samples were tested positive using TBEV ELISA IgM and/or IgG antibody testing (including five borderline results). We identified positive samples in six counties. The total number of sera from the six counties with positive TBEV results was 796. The positive ELISA TBEV antibodies in the six counties represented 3.2%. From these 796 sera, we found 15 (1.9%) IgG TBEV positive samples, 6 (0.7%) IgM positive samples and 5 (0.6%) borderline results. In Table 2, we included the sample size calculated retrospectively for 95% CI and +/−3% error for each county, taking into account the ELISA TBEV results.

From the twenty-six positive individuals, eight reported having a risky profession, and five reported mushroom collecting as their usual activity. Only four remembered a tick bite in their medical history. Compared to the negative patients from the same counties, there were no statistically significant differences regarding risk factors for TBEV.

### 3.3. Serum Neutralization Test Results

For confirmation of ELISA, 26 serum samples with ≥9.0 VE were further tested using a neutralization assay. Of the successfully tested samples, seven samples showed a positive serum neutralization test result. All positive samples were from one site in Sibiu County. Although the overall seroprevalence for Sibiu County was 4.9%, because all the positive samples were from one locality, the seroprevalence was 9.72% CI (4–19). We did not find any significant difference regarding potential risk factors for TBEV infection (see Table 3). The overall study seroprevalence of TBEV antibodies confirmed by neutralization was 0.62%.

## 4. Discussion

In our serosurvey, we analyzed 1116 serum samples from 15 localities in 10 Romanian counties, which were divided into endemic counties, border counties and non-endemic counties. The study population was from rural areas, with a predominance of female participants and a median age of 58. 

The overall seroprevalence was 0.62%. After the TBEV neutralization assay, all our positive samples (7/1116) were centered at one site in Sibiu County. Although the overall seroprevalence for Sibiu County was 4.9%, because all the positive samples were from one locality, the seroprevalence at this site was 9.7%. 

Neutralization assays are the most type-specific serological tests and are recommended for confirmation of TBEV ELISA results, especially in surveys conducted in non-endemic TBEV areas [13]. 

Cases in Sibiu County have been previously reported. Thus, analyzing reported cases of TBEV in Romania, a collaboration project between the VENICE II project and the ECDC that evaluated surveillance of TBE in 17 participating countries from the EU mentioned a number of 67 cases of TBE reported in Romania in 2007, most of them related to a TBE outbreak, also in Sibiu County [14].

Between 2008 and 2014, a passive surveillance system based on reported cases, at a national level, of meningitis, encephalitis, meningoencephalitis, encephalomyelitis and encephalo-radiculitis tested for TBEV was implemented in the north-western counties of Romania, around Cluj (Alba, Bihor, Bistrita-Nasaud, Cluj, Covasna, Harghita, Mures, Maramures, Satu Mare, Salaj, Sibiu, Arad, Hunedoara, Caras-Severin). Only a total of 25 cases were reported during this period, most of them from a few counties. Between 2016 and 2018, five more cases were reported [15]. As a notifiable disease in all countries of the European Union, in the last report, in 2020, 3817 TBE cases were reported to TESSy from EU/EEA countries, 3734 (97.8%) of which were confirmed (0.9 cases per 100,000 population), representing an increasing trend in the EU compared with 2019. Nevertheless, three countries, including Romania, reported no cases [16], which might express the lack of awareness and testing in those countries rather than the lack of infection. 

A study published in 2022 regarding TBEV seroprevalence rates in blood donors from North-West Romania tested 1200 samples from six counties (Alba, Cluj, Salaj, Bistrita-Nasaud, Maramures, Satu-Mare) and reported a seroprevalence of only 0.08% (one positive case after neutralization assay out of the 200 samples from a county under passive surveillance—Satu Mare; the patient resided in a rural area) [17]. Similar studies performed in other European countries, like Norway, found that from 1136 blood donor samples in two counties considered endemic (with at least one positive case reported), the seroprevalence was 0.4% (4/1123) in individuals that had previously undergone TBEV infection, taking into consideration also the existence of vaccinated individuals, with a total seroprevalence of 1.5% (17/1123) [18].

On the other hand, it should be noted that TBEV occurs with natural focus in so-called TBEV microfoci, small areas with a medium size of about 0.5 to 1 ha, where the transmission cycle occurs in the presence of the vector, *I. ricinus,* and the reservoir host (small rodents). These TBEV microfoci are usually traced in nature based on detailed medical histories of TBE patients [19].

Even in seroprevalence studies conducted strictly in populations considered at higher risk for TBE, such as forestry workers from different regions of Northern France, the seroprevalence found was only 0.14% (3/1777). Regions considered hot spots for TBEV like Alsace had no positive results, and new areas like the Franche-Comte region had positive results, raising the question of whether the virus is present over a large area of the country [20]. 

The detection of positive serum samples in serosurvey and seroprevalence studies seems dependent on finding TBEV geographical microfoci. Testing around these specific areas may possibly give a false impression of the absence of the virus in a region. 

The incidence rate and implied identification of new natural foci are strongly dependent on national diagnostic guidelines and diagnostic resources [21]. Patients presenting to the hospital with a confirmed TBEV infection may play a role in detecting new natural foci, and a precise history of the medical case may generate a trace to the origin of the infection and the detection of new areas of interest. But this requires diagnostic tools and medical awareness.

Serological surveys are useful in identifying other potential cases and their related contacts. Furthermore, the situation should be continuously monitored to advise residents and national/foreign travelers to take precautions and prevent the spread of TBEV infections in close contact with TBEV foci. 

Our results cannot be extrapolated as seroprevalence to the level of the entire county where the research was carried out because the collected samples came from at most three localities in the respective county, a small proportion of the total adult population. A serosurvey study is an indicator of exposure, disease burden and immunity to TBEV and is useful in signaling the existence of TBEV foci. Other limitations of our study are related to the sampling protocol and methodology. The localities where the samples were collected were those connected with the program of the mobile unit collecting blood samples for basic blood tests. Thus, we had more localities from some counties, depending on their calendar and our difficulty reaching different remote localities.

The positive ELISA IgG/IgM samples that tested negative in the neutralization assay could have been tested using the indirect immunofluorescence assay for other flaviviruses, especially the West Nile virus (WNV), which is endemic in Romania, while other flaviviruses like the yellow fever virus (YFV), the Japanese encephalitis virus (JEV) or the dengue virus are less likely to be involved in a cross-reactivity. These viruses are most probably linked to travel in endemic areas, which is a low probability for the population studied [13]. Participants included in this study were not vaccinated against YFV and JEV. Unfortunately, we did not test the samples for WNV.

It was recognized that TBE cases in Romania may be misdiagnosed as West Nile encephalitis and vice versa because of antibody cross-reactivity in ELISA assays and the larger scale of WNV testing compared with TBEV testing [22].

## 5. Conclusions

Although the number of samples processed in the present study may not be sufficient for a robust epidemiological assessment of the TBEV infection, our results showed the presence of TBEV infection in Romania, especially in Sibiu County. This study emphasizes the importance of accurate diagnostic tools, awareness and surveillance for effective prevention and control and suggests that further serological surveys should be developed, especially after the identification of positive TBEV patients presenting for medical care.

## Figures and Tables

**Table 1:** Characteristics of the study population by county and the presence of ELISA TBEV antibodies.

Study Population and Counties N (%)	Total N = 1116	Teleorman N = 56 (5)	Brasov N = 96 (8.6)	Alba N = 212 (19.0)	Sibiu N = 143 (12.8)	Suceava N = 182 (16.3)	Botosani N = 82 (7.3)	Constanta N = 74 (6.6)	Prahova N = 67 (6.0)	Hunedoara N = 111 (9.9)	Neamt N = 93 (8.3)
Male sex N (%) (95% CI)	291 (26) (23.5–28.8)	15 (26.8)	14 (14.6)	69 (32.5)	38 (26.6)	59 (32.4)	16 (19.5)	13 (17.6)	21 (31.3)	27 (24.3)	19 (20.4)
Median age (IQR) (years)	58 (47–70)	65 (51–75)	54 (40–69)	60 (51–70)	57 (49–66)	56 (44–67)	56 (46–69)	47 (42–61)	53 (41–69)	59 (50–69)	70 (59–76)
Profession at risk N (%) ^1^ (95% CI)	230 (20.6) (18.3–23.1)	10 (17.9)	4 (4.2)	66 (31.1)	7 (4.9)	58 (31.9)	17 (20.7)	10 (13.5)	8 (11.9)	8 (7.2)	42 (45.2)
Mushroom collector N (%) (95% CI)	266 (23.8) (21.4–26.5)	1 (1.8)	31 (32.3)	73 (34.4)	3 (2.1)	101 (55.5)	2 (2.4)	6 (8.1)	4 (6.0)	29 (26.1)	16 (17.2)
Consumption of unprocessed milk products N (%) (95% CI)	561 (50.3) (47.3–53.3)	22 (39.3)	51 (53.1)	131 (61.8)	54 (37.8)	112 (61.5)	33 (40.2)	41 (55.4)	34 (50.7)	21 (18.9)	62 (66.7)
Tick bite history N (%) (95% CI)	274 (24.5) (22.1–27.2)	4 (7.1)	26 (27.1)	98 (46.2)	26 (18.2)	30 (16.5)	7 (8.5)	12 (16.2)	5 (7.5)	41 (36.9)	25 (26.9)
Domestic animals in household N (%) ^2^ (95% CI)	139 (12.4) (10.6–14.5)	12 (21.4)	13 (13.5)	18 (8.4)	12 (8.4)	17 (9.3)	34 (41.4)	1 (1.4)	5 (7.5)	14 (12.6)	13 (14.0)
ELISA TBEV+ samples N (%) (95% CI)	26 (2.3) (1.5–3.4)	0	0	3 (1.4)	10 (6.9)	5 (2.7)	3 (3.6)	0	3 (4.4)	2 (1.8)	0

^1^—profession at risk = hunter, forest ranger, farmer, leather manufacturer; ^2^—domestic animals = sheep, goats, horses; CI—confidence interval.

**Table 2:** ELISA testing in each county and studied site.

County	Serum Sample N	ELISA TBEV %	Calculated Sample Size	Locality	Serum Samples N (%)	ELISA TBEV+ Samples N (%); 95% CI
Sibiu	142	7	278	Mosna	72 (6.4)	9 (12.5); (5.9–22.4)
				Bazna	71 (6.3)	1 (1.4); (0–7.6)
Brasov	96	0	43	Augustin	44 (3.9)	0; (0–8)
				Poiana Marului	52 (4.6)	0; (0–6.8)
Alba	212	1	43	Rosia de Secas	50 (4.5)	3 (6.0); (1.3-16.5)
				Albac	93 (8.3)	0; (0–3.9)
				Salciua	69 (6.1)	0; (0–5.2)
Suceava	182	2.5	104	Zamostea	100 (8.9)	4 (4.0); (1.1–9.9)
				Valea Moldovei	82 (7.3)	1 (1.2); (0–6.6)
Botosani	82	3	125	Vlasinesti	82 (7.3)	3 (3.6); (0.8–10.3)
Teleorman	56	0	43	Negrenii de Sus	56 (5.0)	0; (0–6.4)
Constanta	74	0	43	Ostrov	74 (6.6)	0; (0–4.9)
Prahova	67	3	125	Colceag	67 (6.0)	3 (4.4); (0.9–12.5)
Hunedoara	111	2	84	Romos	111 (9.9)	2 (1.8); (0.2–6.4)
Neamt	93	0	43	Ghindaoani	93 (8.3)	0; (0–3.9)
				**Total**	**1116 (100)**	**26 (2.3); (1.5–** **3.4)**

**Table 3:** Characteristics of the study population associated with the presence of TBEV antibodies (Mosna/Sibiu).

	Positive TBE N = 7	Negative TBE N = 65	OR (95% CI) *p* Value
Male sex N (%)	2 (28.6)	16 (24.6)	1.22 (0.21–6.93) 0.81
Age Median (IQR)	59 (53–65)	60(51-67)	0.57
Profession at risk N (%)	1 (14.3)	4 (6.2)	2.54 (0.24–26.55) 0.42
Mushroom collector N (%)	0	3 (4.6)	1.11 (1.02–1.20) 0.56
Consumption of unprocessed milk products N (%)	2 (28.6)	23 (35.4)	0.73 (0.13–4.06) 0.71
Tick bite history N(%)	0	14 (21.5)	1.13 (1.03–1.25) 0.17
Domestic animals in household N (%)	0	8 (12.3)	1.12 (1.03–1.22) 0.32
History of recreational activities in nature N (%)	0	2 (3.1)	1.11 (1.02–1.20) 0.63
Knowledge about diseases spread by ticks N (%)	3 (42.9)	50 (76.9)	0.22 (0.04–1.1) 0.05

## Data Availability

The original contributions presented in the study are included in the article, further inquiries can be directed to the corresponding author.

## References

[1] Lindquist L., Vapalahti O. (2008). Tick-borne encephalitis. Lancet.

[2] Mihalca A.D., Gherman C.M., Magdaş C., Dumitrache M.O., Györke A., Sándor A.D., Domşa C., Oltean M., Mircean V., Mărcuţan D.I. (2012). Ixodes ricinus is the dominant questing tick in forest habitats in Romania: The results from a countrywide dragging campaign. Exp. Appl. Acarol..

[3] Topp A.K., Springer A., Mischke R., Rieder J., Feige K., Ganter M., Nagel-Kohl U., Nordhoff M., Boelke M., Becker S. (2023). Seroprevalence of tick-borne encephalitis virus in wild and domestic animals in northern Germany. Ticks Tick Borne Dis..

[4] Ionescu L., Alexse A., Ceianu C., Necsulescu M., Popescu D., Bicheru S., Dumitrescu G., Cumpănăşoiu C.E., Cumpănăşoiu C., Pasat L. (2009). Investigation methods- used for identifing the presence of tick-borne encephalitis virus (TBEV) in vector arthropods. Sci. Pap. Vet. Med..

[5] Vladimirescu A., Dumitrescu G., Ionescu L., Necsulescu M., Moraru V., Popescu D., Bicheru S., Doina D., Baraitareanu D., Ciulacu-Purcarea V. (2016). Real-Time PCR studies regarding the borrelia burgdorferi, francisella tularensis, tick borne encephalitis virus (TBEv) and crimeean congo hemorrhagic fever virus (CCHFv) occurrence in the Romanian ticks. Int. J. Infect. Dis..

[6] Salat J., Mihalca A.D., Mihaiu M., Modrý D., Ruzek D. (2017). Tick-Borne Encephalitis in Sheep, Romania. Emerg. Infect. Dis..

[7] Suss J. (2008). Tick-borne encephalitis in Europe and beyond--the epidemiological situation as of 2007. Eurosurveillance.

[8] Molnár G.B., Persecă T., Feder A., Păcuraru D., Marialaki E., Cojan A. (2008). Evaluarea epidemiologică a emergenţei morbidităţii si focalităţii naturale cu virusul TBE-CEE in Transilvania [Epidemiological assessment of morbidity and natural foci of TBE-CEE virus infection in Transylvania]. Rev. Med. Chir. Soc. Med. Nat. Iasi..

[9] Romanian National Center for Suerveillance of Transmitable Diseases—Suerveillance System for TBEV Infection (Centrul National de Supraveghere a Bolilor Transmisibile—Sistemul de Supraveghere al Infectiei cu Virusul Encefalitei Transmise de Capusa). https://www.cnscbt.ro/index.php/metodologii/tbe/477-tbe-metodologie/file.

[10] Chitimia-Dobler L., Hristea A., Erber W., Jankovic T.V., Dobler G., Erber W., Broker M., Schmitt H.J. (2019). TBE in Romania Chapter12b. The TBE Book.

[11] Ganbold D., Uudus B., Nyamdavaa N., Chultemsuren Y., Zagd A., Tangad M., Badrakh B., Baldandorj B., Dogsom O., Lkunrev R. (2023). Seroprevalence and risk factors of tick-borne encephalitis in Mongolia between 2016 and 2022. Parasite Epidemiol. Control.

[12] VIROTECH FSME/TBE IgG/IgM ELISA—Gebrauchsanweisung/Instruction for Use (IFU). http://ifudownload.virotechdiagnostics.com/ELISA/EC117.00%20VIROTECH%20FSME_TBE%20IgGIgM%20ELISA/02%20EN/VIROTECH%20FSME,TBE%20IgG_IgM%20ELISA%20EN%20REV22.pdf.

[13] Holzmann H. (2003). Diagnosis of tick-borne encephalitis. Vaccine.

[14] VENICE II (2009). Tick-Borne Encephalitis Surveillance Systems and Vaccination Recommendations in UE/EEA. Collaboration between VENICE II Project and ECDC. http://venice.cineca.org/final_report_TBE_19-01-2011.pdf.

[15] European Centre for Disease Prevention and Control (2019). Tick-borne encephalitis. ECDC. Annual Epidemiological Report for 2017.

[16] European Centre for Disease Prevention and Control (2022). Tick-borne encephalitis. ECDC. Annual Epidemiological Report for 2020.

[17] Coroian M., Mihalca A.D., Dobler G., Euringer K., Girl P., Borșan S.D., Kalmár Z., Tincuța Briciu V., Flonta M., Topan A. (2022). Seroprevalence Rates against West Nile, Usutu, and Tick-Borne Encephalitis Viruses in Blood-Donors from North-Western Romania. Int. J. Environ. Res. Public Health.

[18] Marvik Å., Tveten Y., Pedersen A.B., Stiasny K., Andreassen Å.K., Grude N. (2021). Low prevalence of tick-borne encephalitis virus antibodies in Norwegian blood donors. Infect. Dis..

[19] Borde J.P., Glaser R., Braun K., Riach N., Hologa R., Kaier K., Chitimia-Dobler L., Dobler G. (2022). Decoding the Geography of Natural TBEV Microfoci in Germany: A Geostatistical Approach Based on Land-Use Patterns and Climatological Conditions. Int. J. Environ. Res. Public Health.

[20] Septfons A., Rigaud E., Bénézet L., Velay A., Zilliox L., Baldinger L., Gonzalez G., Figoni J., de Valk H., Deffontaines G. (2023). Seroprevalence for *Borrelia burgdorferi* sensu lato and tick-borne encephalitis virus antibodies and associated risk factors among forestry workers in northern France, 2019 to 2020. Eurosurveillance.

[21] Jakimovski D., Mateska S., Dimitrova E., Bosilkovski M., Mijatović D., Simin V., Bogdan I., Grujić J., Budakov-Obradović Z., Meletis E. (2023). Tick-Borne Encephalitis Virus and *Borrelia burgdorferi* Seroprevalence in Balkan Tick-Infested Individuals: A Two-Centre Study. Pathogens.

[22] Kunze M., Banović P., Bogovič P., Briciu V., Čivljak R., Dobler G., Hristea A., Kerlik J., Kuivanen S., Kynčl J. (2022). Recommendations to Improve Tick-Borne Encephalitis Surveillance and Vaccine Uptake in Europe. Microorganisms.

